# Knockdown of ZBTB11 impedes R‐loop elimination and increases the sensitivity to cisplatin by inhibiting DDX1 transcription in bladder cancer

**DOI:** 10.1111/cpr.13325

**Published:** 2022-08-26

**Authors:** Lei Chen, Zefu Liu, Huancheng Tang, Zhaohui Zhou, Jiawei Chen, Zikun Ma, Minhua Deng, Xiangdong Li, Yuanzhong Wu, Lisi Zheng, Liwen Zhou, Xianchong Zheng, Zhuowei Liu

**Affiliations:** ^1^ State Key Laboratory of Oncology in South China, Collaborative Innovation Center for Cancer Medicine Sun Yat‐sen University Cancer Center Guangzhou China; ^2^ Department of Urology Sun Yat‐sen University Cancer Center Guangzhou China

## Abstract

**Introduction:**

Bladder cancer (BC) is one of the most common malignant cancers, with poor prognosis and high incidence. Cisplatin is the standard chemotherapy for muscle invasive bladder cancer; however, chemotherapy resistance remains a major challenge. Moreover, oncogenic signalling and the specific mechanisms underlying cisplatin resistance in BC remain largely unclear

**Methods:**

In this study, RT‐PCR, Western blot, immunofluorescence, and immunohistochemistry were used to measure gene and protein expression. Colony formation assay and flow cytometry were performed to evaluate the proliferation of BC cells. Gene set enrichment analysis was performed to identify the function in which ZBTB11 was involved. Luciferase and chromatin immunoprecipitation experiments were performed to determine the transcriptional regulation mechanism of ZBTB11. The effects of ZBTB11 on the malignant phenotypes of BC cells were examined in vitro and in vivo

**Results:**

The results showed that ZBTB11 was remarkably upregulated in BC tissues, which was associated with poor prognosis in patients with BC. Furthermore, we found that knockdown of ZBTB11 remarkably inhibited the proliferation and tumorigenesis of BC cells by inducing apoptosis. Mechanistically, the knockdown of ZBTB11 transcriptionally inhibited DDX1 to suppress R‐loop clearance, resulting in DNA damage in BC cells. Importantly, the ZBTB11/DDX1 axis is required for the chemotherapy resistance of BC cells to cisplatin

**Conclusion:**

Our findings not only reveal an underlying mechanism by which the ZBTB11/DDX1 axis promotes the tumorigenesis of BC but also provide a potential target for a combination strategy of cisplatin‐based chemotherapy for BC.

## Graphical abstract

In bladder cancer, ZBTB11 promotes the transcription of DDX1, an RNA helicase required for R‐loop clearance. Therefore, knockdown of ZBTB11 leads to DNA damage as a result of R‐loop accumulation, which further reduces proliferation, promotes apoptosis and increases cisplatin sensitivity of bladder cancer cells.

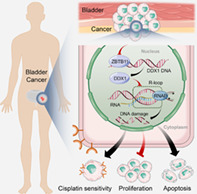

## 1 INTRODUCTION

Bladder cancer (BC) is the sixth most common cancer worldwide; its mortality accounts for 3% of cancer‐related deaths.^1^ BC is conventionally classified as muscle invasive bladder cancer (MIBC) or non‐MIBC depending on whether or not it is invasive.^2^ Non‐MIBC often recurs; therefore, patients require long‐term disease monitoring, which increases medical expenses.^3^ MIBC has a poor prognosis owing to spread and occult metastasis.^4^ Clinically, the cisplatin‐based combined strategy of gemcitabine plus cisplatin has become the standard treatment for MIBC.^4^, ^5^, ^6^, ^7^ Almost 60% of patients with metastatic MIBC show an effective response to cisplatin‐based strategies; however, chemotherapy resistance remains a major challenge.^4^, ^5^ Immune checkpoint inhibitors have raised new hope; however, the five‐year survival rate of MIBC is still less than 50%.^4^ Finding new targets for inhibiting BC progression and chemotherapy resistance is an important area of cancer research.

The abnormal regulation of transcription factors is an important hallmark of tumour growth. The zinc‐finger and BTB domain‐containing (ZBTB) family of proteins are nuclear transcription factors that directly regulate the transcription of multiple genes.^8^ Certain ZBTB genes have been identified as important oncogenes (e.g., BCL6/ZBTB27 and ZBTB7A^9^), whereas some have been identified as tumour suppressors (e.g., HIC1/ZBTB29^10^). High expression of BCL6 protein is associated with the pathogenesis of lymphoma and ovarian cancer.^11^, ^12^ Recent studies have implicated it as an effective cancer therapeutic target.^13^, ^14^, ^15^, ^16^ In our previous study, we screened the CRISPR–Cas9 transcription factor library and found that ZBTB11 is the only protein of the ZBTB family that plays a crucial role in the proliferation of BC cell lines. Recently, ZBTB11 has been associated with intellectual disability‐associated factor and has been reported to regulate neutrophil development and mitochondrial function.^17^, ^18^ Previous studies using chromatin immunoprecipitation sequencing have demonstrated that ZBTB11 can specifically recognize and bind to the ‘CGGAA’ motif in the promoter region of downstream genes to regulate transcription.^18^ Inactivation of the ZBTB11 gene results in impaired mitochondrial respiratory function, ultimately leading to proliferation arrest and cell death.^18^ Thus, we speculated that ZBTB11 plays a role in controlling the proliferation of BC cells. R‐loop is a special nucleic acid structure that is composed of a double‐stranded RNA–DNA hybrid and non‐template, single‐stranded DNA.^19^ R‐loop is closely related to physiology and participates in the progression of various tumours.^20^, ^21^ Excessive R‐loop accumulation in cancer cells induces DNA damage, leading to genomic instability and apoptosis.^22^, ^23^ It is plausible that targeting R‐loop clearance mechanisms could make tumours more vulnerable to DNA‐damaging drugs, such as cisplatin. For example, the accumulation of R‐loop and increased sensitivity to cisplatin can be achieved in synovial sarcoma and Ewing's sarcoma by the clinical use of ataxia telangiectasia‐mutated and Rad3‐related (ATR) kinase inhibitors.^24^ Furthermore, luteolin can lead to the formation of R‐loop and subsequently damage DNA; in hepatocellular carcinoma, it confers sensitivity to cisplatin by targeting THOC1.^25^ It is known that excessive R‐loop in cancer cells can be recognized and degraded by RNA enzymes (e.g., RNAse H1). However, the R‐loop clearance mechanism in cancer cells is largely unknown.^26^ This study aimed to investigate the oncogenic role of ZBTB11 in BC cells in vitro and in vivo. Our results demonstrated that ZBTB11 knockdown transcriptionally inhibited DDX1 to accumulate R‐loop and induce DNA damage in BC cells. Importantly, we also determined that knockdown of ZBTB11 in BC cells suppressed their proliferation and increased their sensitivity to cisplatin by downregulating DDX1. Our findings indicate that the ZBTB11/DDX1 axis may be a promising therapeutic target for cisplatin‐based chemotherapy in BC

## 2 MATERIALS AND METHODS

### 2.1 Patients and tissue specimens

BC samples (*n* = 130) were obtained from patients who underwent radical resection for BC at the Sun Yat‐sen University Cancer Center (SYSUCC). The samples were collected and paraffin‐embedded at SYSUCC from 2008 to 2016. Each sample contained BC tumour tissue and paired normal bladder tissue. None of the patients had received any antitumor therapy prior to biopsy. The Ethics Review Committee of SYSUCC approved the study. Informed consent was obtained from all patients. The tumour–node–metastasis staging system (2017, 8th edition), jointly developed by the American Joint Committee on Cancer and the Union for International Cancer Control, was used for clinical staging.

### 2.2 Immunohistochemical analysis

Immunohistochemically‐stained samples were cut into 5‐μm cross‐sections and placed on slides that were dehydrated before the analysis. After blocking with 10% normal goat serum for 20 min, the sections were incubated at 4°C overnight with the primary antibody anti‐ZBTB11 (1:200, Thermofisher, Waltham, MA, Cat. No. PA5‐54992). The sections were then incubated with horseradish peroxidase‐conjugated secondary antibody at room temperature for 1 h, stained with 3,3‐diaminobenzidine solution, and counterstained with haematoxylin. The sample images were acquired using an automatic slide scanner (KFBIO, Zhejiang, China). To evaluate the expression level of ZBTB11, we used HALO pathology analysis software (Indica Labs, Albuquerque, NM) with a multiplex immunohistochemistry module.

### 2.3 Cell lines and cell culture

Human BC cell lines T24 and UMUC3 and human embryonic kidney cells HEK293T were purchased and authenticated from the American Type Culture Collection (Waltham, MA). Cell lines were authenticated 3 months before study initiation based on their viability and morphology. All cell lines were cultured at 37°C in 5% CO_2_ in high‐glucose Dulbecco's Modified Eagle Medium (Gibco, Waltham, MA) supplemented with 10% foetal bovine serum (Gibco), penicillin (100 units/ml), and streptomycin (100 μg/ml).

### 2.4 Plasmid construction

The human complementary DNAs (cDNA) of ZBTB11, DDX1 and RNase H1(N‐terminal SFB‐tagged ZBTB11, FLAG‐tagged ZBTB11, HA‐tagged ZBTB11, and HA‐tagged RNase H1) were cloned into pSIN‐EF1α‐puro vector. The short hairpin RNA (shRNA)‐insensitive ZBTB11 expression vector was generated by synonymous mutation of the shRNA binding site and replacement of the puromycin selection marker with a blasticidin cassette in pSIN‐EF1α‐puro vector. The promoter region of DDX1 and its indicated arginine mutations were cloned into the pGL3 vector. The LentiCRISPR‐V2‐puro vector was used to clone single guide RNAs (sgRNAs); the PLKO.1‐puro vector was used to clone shRNAs. The sgRNA and shRNA sequences are shown in Table S1.

### 2.5 Packaging and infection of lentivirus

Stable expression of sgRNA or shRNA was obtained by lentiviral transduction of T24 and UMUC3 cells using the LentiCRISPR‐V2‐puro or pLKO.1‐puro system, respectively. The expression of ZBTB11 in ZBTB11 knockdown BC cells was rescued by expressing the shRNA‐insensitive ZBTB11. Lentivirus was packaged by co‐transfected target plasmids having the PSA/PIG system in 293T cells using a polyethylenimine transfection reagent according to the manufacturer's instructions (Polysciences, Warrington, PA). T24 and UMUC3 cells were resuspended in viral supernatants and mixed with polybrene 8 mg/ml (Sigma–Aldrich, St. Louis, MO) and cultured for 2 days for infection. After lentiviral infection, stable expression cell lines were selected for at least 4 days using 2 mg/ml puromycin or 5 mg/ml blasticidin (Sigma–Aldrich).

### 2.6 Western blot analysis

At the indicated time points, cells were collected, lysed in SDS buffer (Beyotime, Shanghai, China), and centrifuged at 4°C for 15 min at 14,000*g*. Proteins (20 μg) were separated using polyacrylamide gels, then transferred to 0.22‐μm poly (vinylidene fluoride) membranes (Merck Millipore, Burlington, MA). The membrane was blocked with 5% bovine serum albumin for 1 h at room temperature. The membrane was incubated with primary antibodies followed by secondary antibody (1:5000, Promega, Beijing, China, Cat. No. W4011/W4021). Detection was then performed using enhanced chemiluminescence detection reagents. Primary antibodies against γH2AX (1:1000, Cell Signaling Technology, Danvers, MA, Cat. No. 9718S), s9.6 (1:2000, Merck Millipore, USA, Cat. No. MABE1095), DDX1 (1:2000, Proteintech, Wuhan, China, Cat. No. 11357‐1‐AP), PARP (1:1000, Cell Signaling Technology, Danvers, MA, Cat. No. 9542S), Cleaved‐PARP (1:1000, Cell Signaling Technology, Cat. No. 9548S), Caspase3 (1:1000, Cell Signaling Technology, Cat. No. 9662S), Cleaved‐Caspase3 (1:1000, Cell Signaling Technology, Cat. No. 9661S), Caspase9 (1:1000, Cell Signaling Technology, Cat. No. 9504S), Cleaved‐caspase9 (1:1000, Cell Signaling Technology, Cat. No. 9509S), Bcl2 (1:1000, Cell Signaling Technology, Cat. No. 15071S), Bax (1:1000, Cell Signaling Technology, Cat. No. 14796S), Flag Tag (1:1000, Cell Signaling Technology, Cat. No. 14793S), HA Tag (1:1000, Cell Signaling Technology, Cat. No. 3724S) were used. GAPDH (1:2000, Proteintech, Cat. No. 60004‐1‐lg) and β‐actin (1:2000, Proteintech, Cat. No. 66009‐1‐lg) were used as internal control.

### 2.7 MTT assay

Cells were seeded in 96‐well plates at a density of 2000 per well and cultured overnight for adherence. MTT solution (Sigma–Aldrich) was added to each well at the indicated time points and then the plates were incubated for another 4 h at 37°C. After removing the medium, formazan crystals were dissolved in dimethyl sulfoxide. MTT absorbance was measured using a spectrophotometer at 490 nm. Relative cell growth was analysed based on MTT absorbance.

### 2.8 Colony formation assay

Cells were trypsinized and seeded in 6‐well plates at a density of 800 cells per well. After continuous culture for 8 days, cell colonies were stained with crystal violet. The number of colonies was analysed.

### 2.9 In vivo xenograft assay

Animal experiments were approved by the Animal Ethics Committee of SYSUCC (No. L102042021010I) and Guidelines for the Care and Use of Laboratory Animals were strictly followed. Five‐week‐old female BALB/c nude mice were purchased from Guangdong Medical Laboratory Animal Center (Guangzhou, China). Wild type or knockdown UMUC3 cells were subcutaneously inoculated into the posterior axilla of the mice (1.5 × 10^6^ cells in 0.1 ml phosphate‐buffered saline [PBS]). The length (*L*) and width (*W*) of the tumour were measured using callipers every 3 days for 3 weeks. Tumour volume was calculated using the following equation: volume (mm^3^) = *L* × *W* ^2^ × 0.5.

### 2.10 RNA‐Seq analysis

RNA‐Seq analysis of ZBTB11 sgRNA and ZBTB11 sgNC cells was performed by Novagene Co. Ltd. (China) using a NovaSeq 6000 system (Illumina, San Diego, CA) with 1 × 10^6^ cells per sample. RNA‐Seq results were aligned with human genome GRCh38 (Hg38) using HISAT2 version 2.0.5 (http://daehwankimlab.github.io/hisat2/).

### 2.11 Real‐time polymerase chain reaction analysis

Total RNA was prepared using an RNA purification kit (Tiangen, Beijing, China) followed by a genomic DNA wipe and reverse transcription using the HiScript III 1st Strand cDNA synthesis kit (Vazyme, Nanjing, China). Real‐time polymerase chain reaction (RT‐PCR) with reverse transcription was performed using a Light Cycler 480 (Roche Diagnostics, Basel, Switzerland) with SYBR Green PCR Master Mix (Vazyme). The 2^−ΔΔCT^ method was used to calculate relative gene expressions. GAPDH was used as the housekeeping gene. The sequences of the primers used are listed in Table S2.

### 2.12 Flow cytometry analysis

After being cultured in 6‐well plates for 2 days, cells were digested and centrifuged with trypsin without ethylenediaminetetraacetic acid. They were then washed twice with ice‐cold PBS (pH: 7.2–7.4) and suspended in binding buffer at 1 × 10^6^ cells per ml. A total of 500 μl of cell suspension was stained for 15 min in darkness at 25°C with 5 μl of Annexin V‐FITC (51‐65874X; BD, Franklin Lakes, NJ) and 5 μl of PI (51‐66211E; BD). Cells were analysed using flow cytometry within 1 h of preparation. The results were analysed using CytExpert Setup 2 software version 4.0.28 (Beckman Coulter Life Sciences, Indianapolis, IN).

### 2.13 Dual luciferase reporter assay

The potential binding sequence or mutation binding sequence was cloned into the pGL3 firefly luciferase reporter vector. UMUC3 cells were seeded in 24‐well plates at a density of 1 × 10^5^ cells per well with 200 ng pGL‐3 reporter gene combined with 20 ng of Renilla luciferase plasmid (pRL‐TK), which was transfected into shNC or shZBTB11 cells and then incubated for 48 h. Passive lysis buffer (Promega, Madison, WI, Cat. No. E1960) was used to prepare cell lysate. A dual luciferase analysis system (Promega, Cat. No. E1960) was used to measure luciferase activity. Luciferase activity was normalized to the activity of the Renilla cell luciferase plasmid.

### 2.14 Chromatin immunoprecipitation

Plus Sonication Chromatin IP Kit (Cell Signaling Technologies, Cat. No. 56383S) was used to prepare chromatin for chromatin immunoprecipitation (ChIP) analysis according to the manufacturer's protocol. After UMUC3 cells were cross‐linked in 1% formaldehyde at room temperature for 10 min, the cross‐linking reaction was terminated by glycine. Cells were collected and lysed, following which chromatin was further shortened to 100–1000 bp using ultrasound. The DNA–protein complex (25 μg) was immunoprecipitated with anti‐FLAG antibody (Cell Signaling Technologies, Cat. No. 14793s) or control rabbit polyclonal anti‐IgG antibody (Cell Signaling Technologies, Cat. No. 2729S). Cross‐links were subsequently de‐crosslinked before DNA extraction. PCR was performed with the DNA product as a template. The DDX1 promoter region was amplified using primers (Table S3).

### 2.15 R‐loop detection using Dot blot

Total nucleic acid from cultured BC cells was extracted using a DNA extraction kit. Indicated amounts of purified nucleic acid were applied to the Hybond N+/positive nylon membranes (RPN303B; GE Healthcare, Chicago, IL), which were placed in Bio Dot apparatus (1706545; Bio‐Rad, Hercules, CA). The membrane was subsequently UV cross‐linked (0.12 J/m^2^) and blocked with 5% milk/tris‐buffered saline with Tween 20 (TBST) for 1 h at room temperature. The level of R‐loops was detected by incubation with mouse monoclonal S9.6 antibody (1:2000; Abcam, Cat. No. ab234957) overnight at 4°C and with secondary antibodies (1:5000, Promega, Cat. No. W4021) at 37°C for 1 h after three washes in TBST. Bands were visualized using an enhanced chemiluminescent detection kit (Thermo Fisher).

### 2.16 Statistical analysis

All experiments were performed in triplicates. Graphs were created and statistical analyses were performed using GraphPad Prism version 9.0 (GraphPad Software). Data are presented as mean ± SD. Statistical significance was calculated using the two‐tailed Student's *t*‐test or analysis of variance test. Survival analysis was performed using the Kaplan–Meier method and log‐rank test. Differences were considered statistically significant at a *p* value of <0.05 (*p < 0.05; **p < 0.01; ***p < 0.001; ****p < 0.0001).

## 3 RESULTS

### 3.1 ZBTB11 is highly expressed in BC tissues and is associated with cancer progression in patients with BC

**FIGURE 1:**
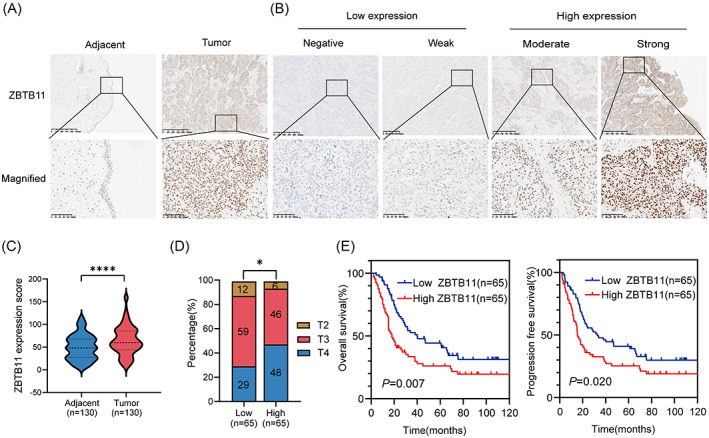
ZBTB11 is upregulated in BC and associated with poor prognosis in patients with BC. (A) Representative immunohistochemical analysis images of ZBTB11 expression in BC tumour and paired adjacent normal tissues. Scale bars: 100 μm. (B) Negative and weak levels of ZBTB11 in BC were considered as low expression; moderate and strong staining intensities were determined as high expression. (C) ZBTB11 expression scores from tumour and paired adjacent normal tissues of BC are presented as a violin plot. Statistical significance was calculated using the two‐tailed Student's *t*‐test. (D) The percentages of BC tissues from T2 to T4 based on the tumour–node–metastasis staging system with low or high expression of ZBTB11 are presented as a bar chart. Statistical significance was calculated using a chi‐square test to compare percentage of T2–T4 BC samples. (E) Survival curves for patients with BC with high or low expression of ZBTB11. Statistical significance was calculated using Kaplan–Meier analysis and the log‐rank test.

To determine whether the expression of ZBTB11 was associated with BC progression, we evaluated the protein levels of ZBTB11 in 130 BC tissues and paired normal bladder tissues using immunohistochemistry. The expression level of ZBTB11 was significantly higher in BC tissues than in paired normal tissues (Figure 1A,C). BC samples were then classified into low ZBTB11 expression and high ZBTB11 expression groups based on immunohistochemistry staining intensity (Figure 1B). Notably, the percentage of T4 BC samples was higher in the high expression group than in the low expression group (Figure 1D). Kaplan–Meier analysis showed that patients with BC with high ZBTB11 expression had poorer overall survival and progression free survival than those with low ZBTB11 expression. These data indicate that high expression of ZBTB11 is associated with BC progression.

### 3.2 ZBTB11 promotes the tumorigenesis of BC cells

**FIGURE 2:**
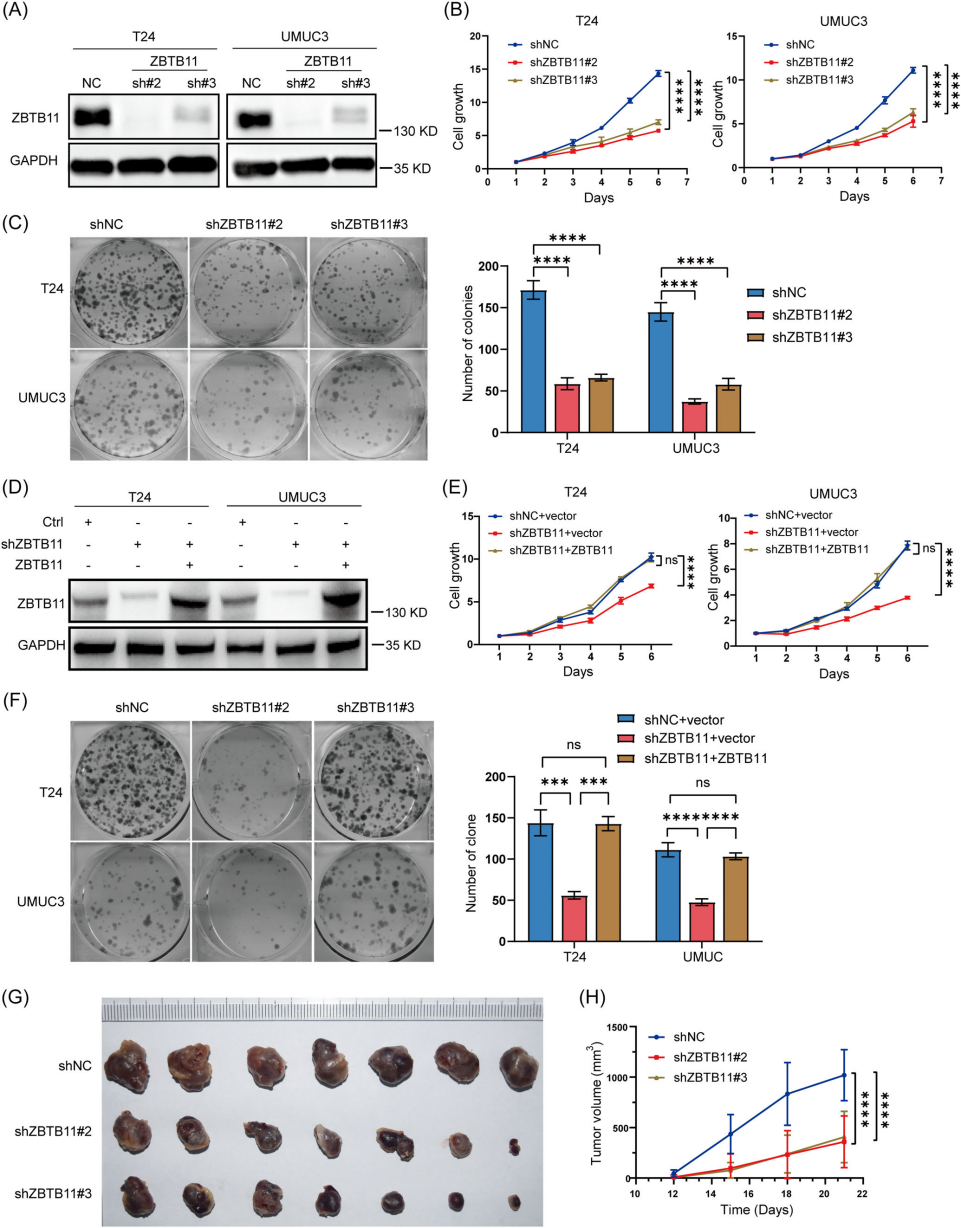
ZBTB11 promotes the proliferation and growth of BC cells. (A) The expression of ZBTB11 in T24 and UMUC3 cells with stable knockdown of ZBTB11 was detected using Western blot analysis. GAPDH was used as an internal control. (B) The proliferation of T24 and UMUC3 cells with stable knockdown of ZBTB11 was evaluated by MTT assay. The relative cell growth curves are presented. Statistical significance was calculated using two‐way analysis of variance. (C) The colony formation ability of stable knockdown BC cells was evaluated by colony formation assay. The numbers of colonies are presented as a bar chart. Statistical significance was calculated using one‐way analysis of variance. (D) Rescue of ZBTB11 expression was achieved by transfecting shRNA‐insensitive ZBTB11 vector into ZBTB11 knockdown BC cells. The expressions of ZBTB11 were detected by Western blot analysis. (E, F) The proliferation and colony formation ability of ZBTB11‐rescued ZBTB11 knockdown BC cells were evaluated by MTT assay and colony formation assay, respectively. The relative cell growth curves and numbers of colonies are shown. (G, H) The tumorigenic ability of UMUC3 cells with ZBTB11 knockdown was assessed by subcutaneous xenograft assay. Images of fixed tumours are presented. Tumour volumes at indicated days were recorded and graphed. Statistical significance was assessed by two‐way analysis of variance.

To investigate the role of ZBTB11 in BC progression, we stably knocked down the expression of ZBTB11 in T24 and UMUC3 cell lines with two independent shRNAs sh#2 and sh#3. Data from Western blot analysis showed that the expression of ZBTB11 was remarkably knocked down in T24 and UMUC3 cells (Figure 2A). Because a previous report suggests that ZBTB11 may be involved in proliferation, we first explored its role in the proliferation of BC cells. Data from the MTT and colony formation assays collectively determined that knockdown of ZBTB11 significantly inhibited the proliferation and colony formation ability of BC cells (Figure 2B,C). However, exogenous expression of ZBTB11 did not significantly influence the proliferation and colony formation ability of BC cells (Figure S1A–C). Knockdown of ZBTB11 suppressed the proliferation of BC cells, whereas the rescue of ZBTB11 expression restored their proliferative ability to the original level (Figure 2D–F). These results were subsequently validated by our finding that subcutaneous xenograft of knockdown ZBTB11 impeded tumour growth and reduced tumour size in UMUC3 cells (Figure 2G,H). These results indicate that the endogenous expression of ZBTB11 is essential for BC tumorigenesis and that additionally expressed ZBTB11 is redundant for BC cell proliferation.

### 3.3 Knockdown of ZBTB11 induces apoptosis in BC cells

**FIGURE 3:**
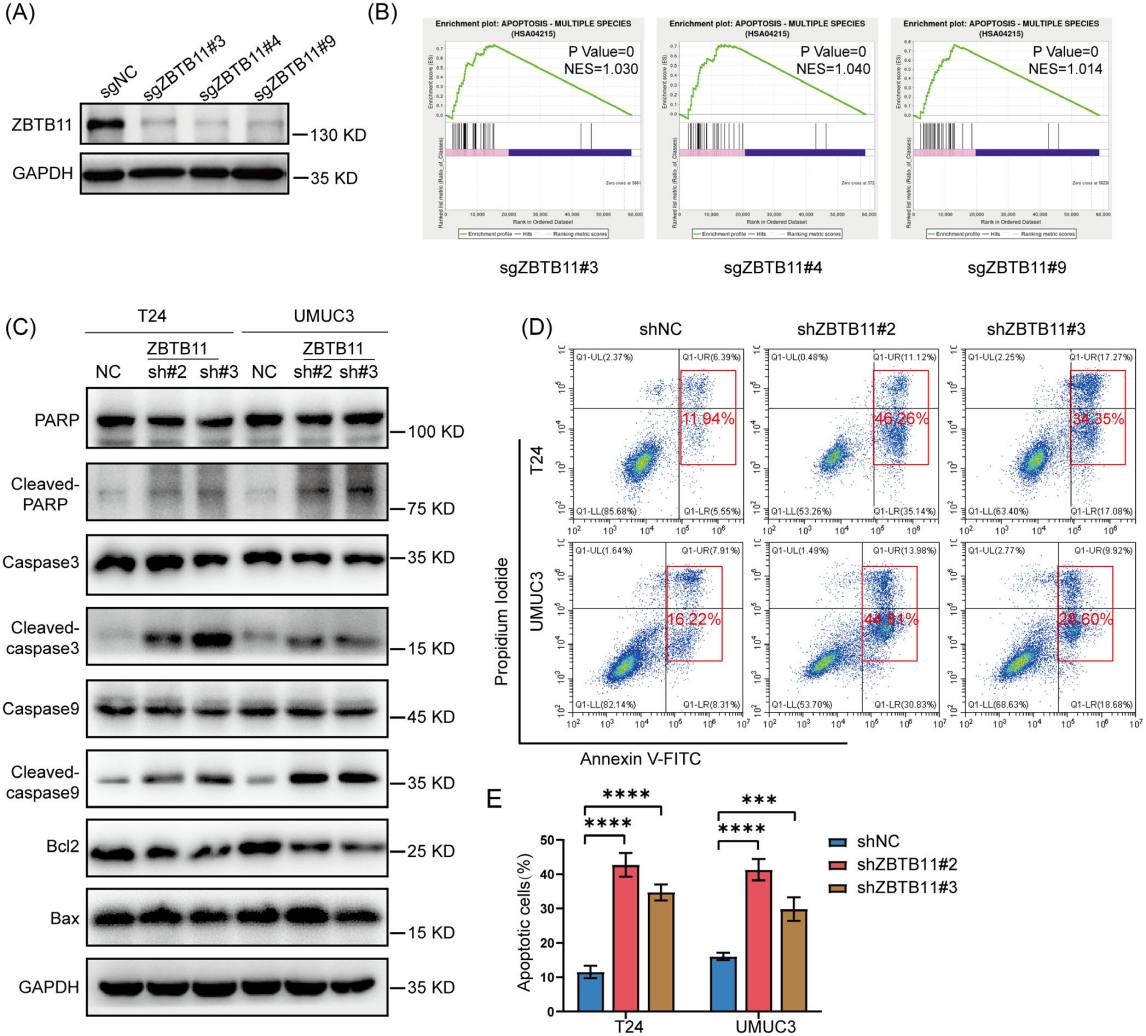
Knockdown of ZBTB11 induces apoptosis in BC cells. (A) The expression of ZBTB11 in T24 cells was knocked down using CRISPR–Cas9 with three independent sgRNAs. The knockdown efficiency was evaluated by Western blot analysis. (B) GSEA plots of apoptotic pathway were analysed from the RNA‐Seq data of T24 cells with ZBTB11 knockdown. (C) The expression of proteins from the apoptotic pathway in ZBTB11 knockdown BC cells was detected by Western blot analysis. (D, E) The apoptosis of ZBTB11 knockdown BC cells was analysed by flow cytometry. The percentages of apoptotic cells are presented as a bar chart. Statistical significance was assessed using one‐way analysis of variance.

To reveal the potential mechanisms of ZBTB11 in BC tumorigenesis, we generated ZBTB11 knockout T24 cells by lentivirus mediated CRISPR–Cas9 and sgRNAs (Figure 3A). At day 4 after lentivirus removal, the ZBTB11 knockout T24 cells were subjected to RNA‐Seq analysis because the proliferation of T24 cells was significantly inhibited at this time (Figure S2A). Data from the gene set enrichment analysis showed that knockout of ZBTB11 with each sgRNA activated the apoptotic pathway in T24 cells (Figure 3B). To verify this finding, we detected the expressions of apoptotic markers in ZBTB11 knockdown BC cells. Data from Western blot analysis showed that knockdown of ZBTB11 in BC cells promoted the cleavage of poly (ADP‐ribose) polymerase, Caspase‐3, and Caspase‐9 and downregulated the expression of Bcl‐2 (Figure 3C). Evidently, knockdown of ZBTB11 promoted apoptosis in BC cells, as evaluated by flow cytometry (Figure 3D,E). These results indicate that knockdown of ZBTB11 induces apoptosis in BC cells.

### 3.4 ZBTB11 activates the transcription of DDX1 in BC cells

**FIGURE 4:**
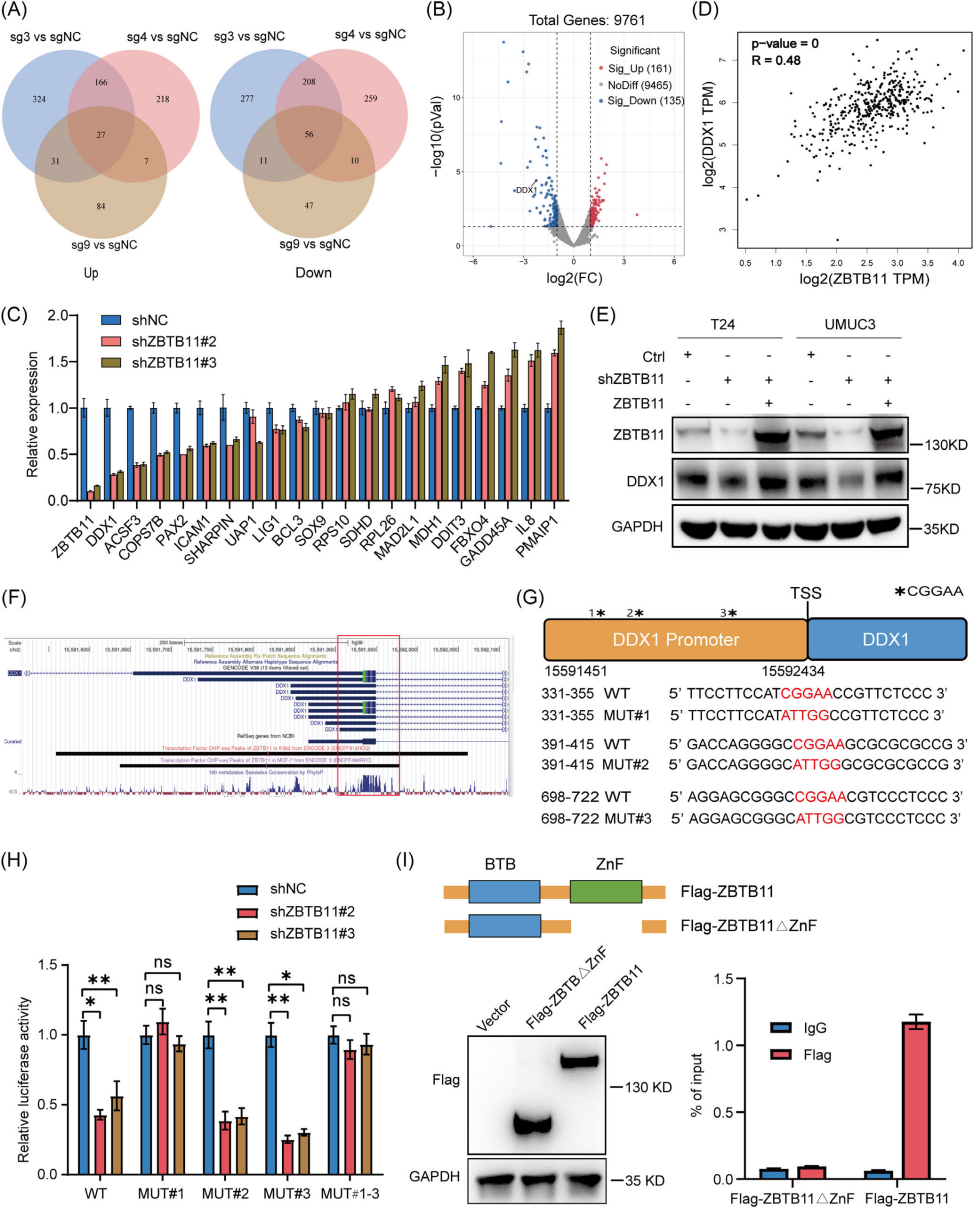
ZBTB11 transcriptionally regulates DDX1 in BC cells. (A) The differential genes from the RNA‐Seq data of ZBTB11 knockout T24 cells are shown in Venn diagrams. (B) The differential genes of ZBTB11 knockout T24 cells are shown as a volcano plot. ZBTB11 knockout significantly downregulated DDX1. (C) mRNA expression of different genes in ZBTB11 knockdown BC cells was evaluated by qRT‐PCR analysis. (D) Correlation between ZBTB11 and DDX1 in BC, as predicted by the GEPIA database (gepia.cancer‐pku.cn), is shown. (E) Protein expression of DDX1 in ZBTB11‐rescued ZBTB11 knockdown BC cells was evaluated by Western blot analysis. (F) The binding peaks of ZBTB11 in the promoter regions of DDX1 in K562 and MCF‐7 cells were visualized using the ENCODE database (www.encodeproject.org). (G) CGGAA‐containing sequences and mutated sequences in DDX1 promoter region. The DDX1 promoter sequence and the mutated sequences were individually cloned into the pGL3 vector for the luciferase reporter assay. (H) The luciferase activities of CGGAA‐containing sequences and of mutated sequences in ZBTB11 knockdown UMUC3 cells were assessed. Relative luciferase activities are presented as a bar chart. Statistical significance was assessed using one‐way analysis of variance. (I) UMUC3 cells were transfected with Flag‐ZBTB11△ZnF plasmids or wild‐type Flag‐ZBTB11 plasmids. Flag expression was detected by Western blot. ChIP qRT‐PCR analysis was performed to confirm whether ZBTB11 binds to the promoter region of DDX1 gene via the ZnF_C2HC domain.

To determine how ZBTB11 knockout induces apoptosis in BC cells, we further analysed the RNA‐Seq data. Our Venn diagrams show that knockout of ZBTB11 significantly upregulated 27 genes and downregulated 56 genes in T24 cells (Figure 4A) and we upload the results of differential gene expression (Table S4). The differential expression of genes caused by ZBTB11 knockout was visualized using a volcano plot (Figure 4B) and heat maps (Figure S2B). Therefore, we investigated the differential expression of the above genes under ZBTB11 knockdown. We used qRT‐PCR to detect gene expression and genes with a CT value of >30 was excluded. Notably, DDX1 was the most significantly downregulated genes after ZBTB11 knockdown (Figure 4C). Among these differential genes, we studied DDX1, which has been highlighted as an oncogene in multiple solid tumours.^27^, ^28^, ^29^, ^30^ By mining The Cancer Genome Atlas data, we found that the mRNA levels of ZBTB11 and DDX1 were positively correlated in BC tissues (Figure 4D). Western blot analysis consistently revealed that knockdown of ZBTB11 drastically downregulated the protein expression of DDX1 in BC cells and that the expression of shRNA‐insensitive ZBTB11 restored the expression of DDX1 (Figure 4E). Using the ENCODE database, we subsequently found binding peaks of ZBTB11 in the promoter regions of DDX1 in human cancer K562 and MCF‐7 cells (Figure 4F). To clarify whether ZBTB11 also binds to the promoter region of DDX1 in BC cells, we cloned the wild‐type DDX1 promoter and a promoter sequence with a mutant ‘CGGAA’ motif into the pGL3 vector (Figure 4G). Notably, the knockdown of ZBTB11 decreased the luciferase activity of the wild‐type reporter in UMUC3 cells (Figure 4H). However, only mutation of the first ‘CGGAA’ motif (MUT#1) in the wild‐type reporter recovered the reduction in luciferase activity in ZBTB11 knockdown UMUC3 cells (Figure 4H). The amino acid domain (569–937) of ZBTB11 contains a zinc‐finger structure (ZnF_C2HC) that is considered to be responsible for DNA binding.^31^ We constructed a Flag‐tagged ZBTB11 expression vector with ZnF_C2HC domain deletion (Flag‐ZBTB11△ZnF) (Figure 4I). ChIP assay was performed to confirm whether ZBTB11 binds to the promoter region of DDX1 gene via the ZnF_C2HC domain. Data from the ChIP assay showed that wild‐type Flag‐ZBTB11, but not Flag‐ZBTB11△ZnF, could significantly bind with the promoter region of DDX1 (Figure 4I). Taken together, these results show that ZBTB11 transcriptionally activates DDX1 by binding to its promoter region in BC cells.

### 3.5 Knockdown of ZBTB11 induces R‐loop accumulation and DNA damage to suppress the growth of BC cells via DDX1 downregulation

**FIGURE 5:**
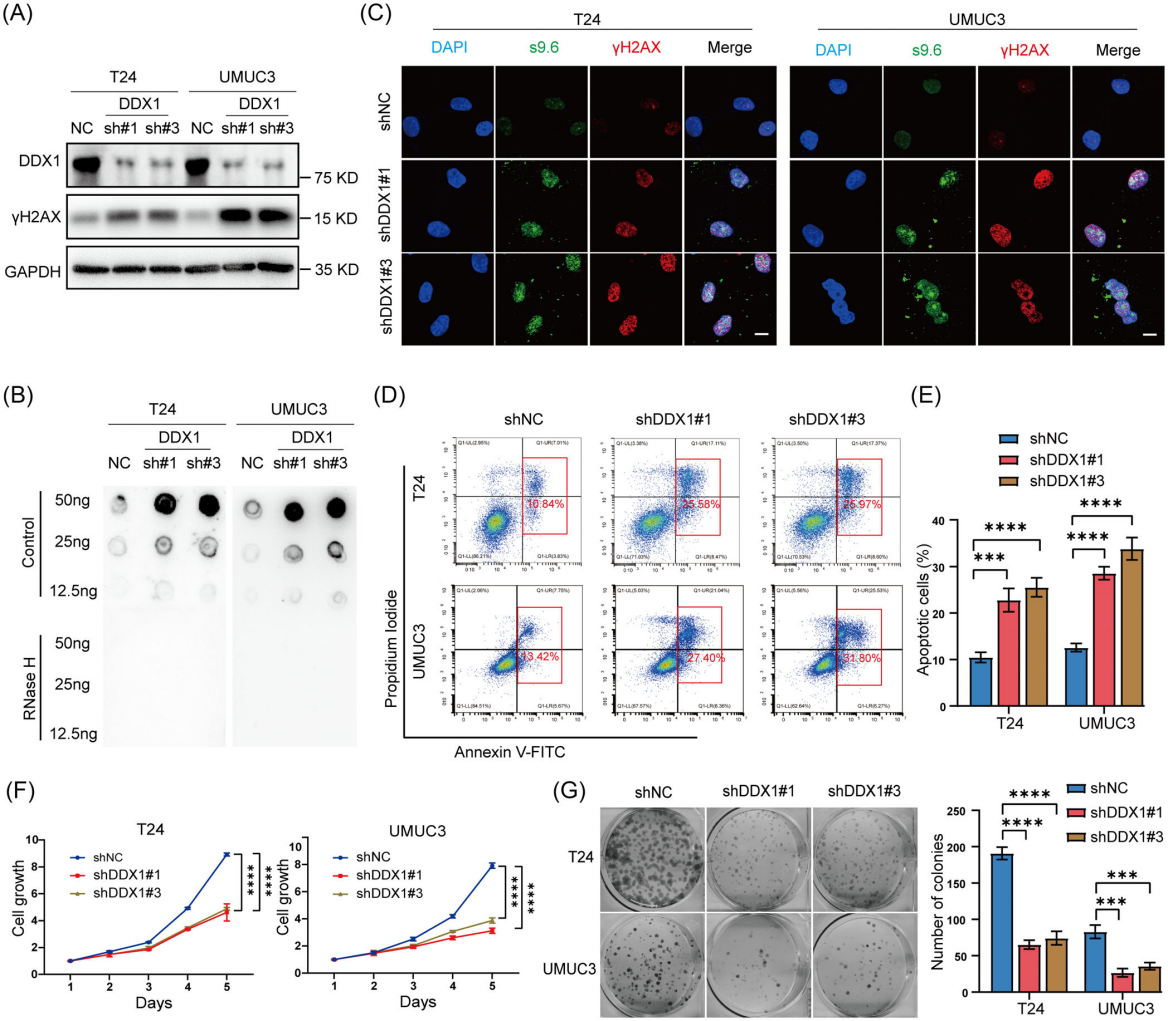
Knockdown of ZBTB11 increases R‐loop accumulation to induce DNA damage and apoptosis in BC cells. (A) The expression of DDX1 in BC cells was knocked down by shRNAs; expressions of DDX1 and γH2AX were detected by Western blot. (B) R‐loop in BC cells with DDX1 knockdown was detected by Dot blot using S9.6 antibody. (C) R‐loop and DNA damage in DDX1 knockdown BC cells were detected by immunofluorescence using antibodies against S9.6 and γH2AX, respectively. Scale bar, 10 μm. (D) The apoptosis of DDX1 knockdown BC cells was analysed using flow cytometry. (E) Percentages of apoptotic cells are presented as a bar chart. Statistical significance was assessed using one‐way analysis of variance. (F, G) The proliferation and colony formation ability of DDX1 knockdown BC cells were evaluated using MTT assay and colony formation assay, respectively. The relative cell growth curves and numbers of colonies are shown. Statistical significance was assessed using one‐way or two‐way analysis of variance.

Although DDX1 is involved in R‐loop clearance at DNA double‐strand breaks,^32^, ^33^ this role has not been established in BC cells. To this end, we established DDX1 stable knockdown BC cells by two independent shRNAs. Data from Western blot analysis showed that knockdown of DDX1 increased the expression of γH2AX, a marker for DNA damage response (Figure 5A). By Dot blot and immunofluorescence analyses, we determined that knockdown of DDX1 increased R‐loop accumulation in BC cells, as shown by increased levels of S9.6^24^ and γH2AX (Figure 5B,C). Similarly, knockdown of DDX1 also induced apoptosis and impeded the growth of BC cells (Figure 5D–G).

**FIGURE 6:**
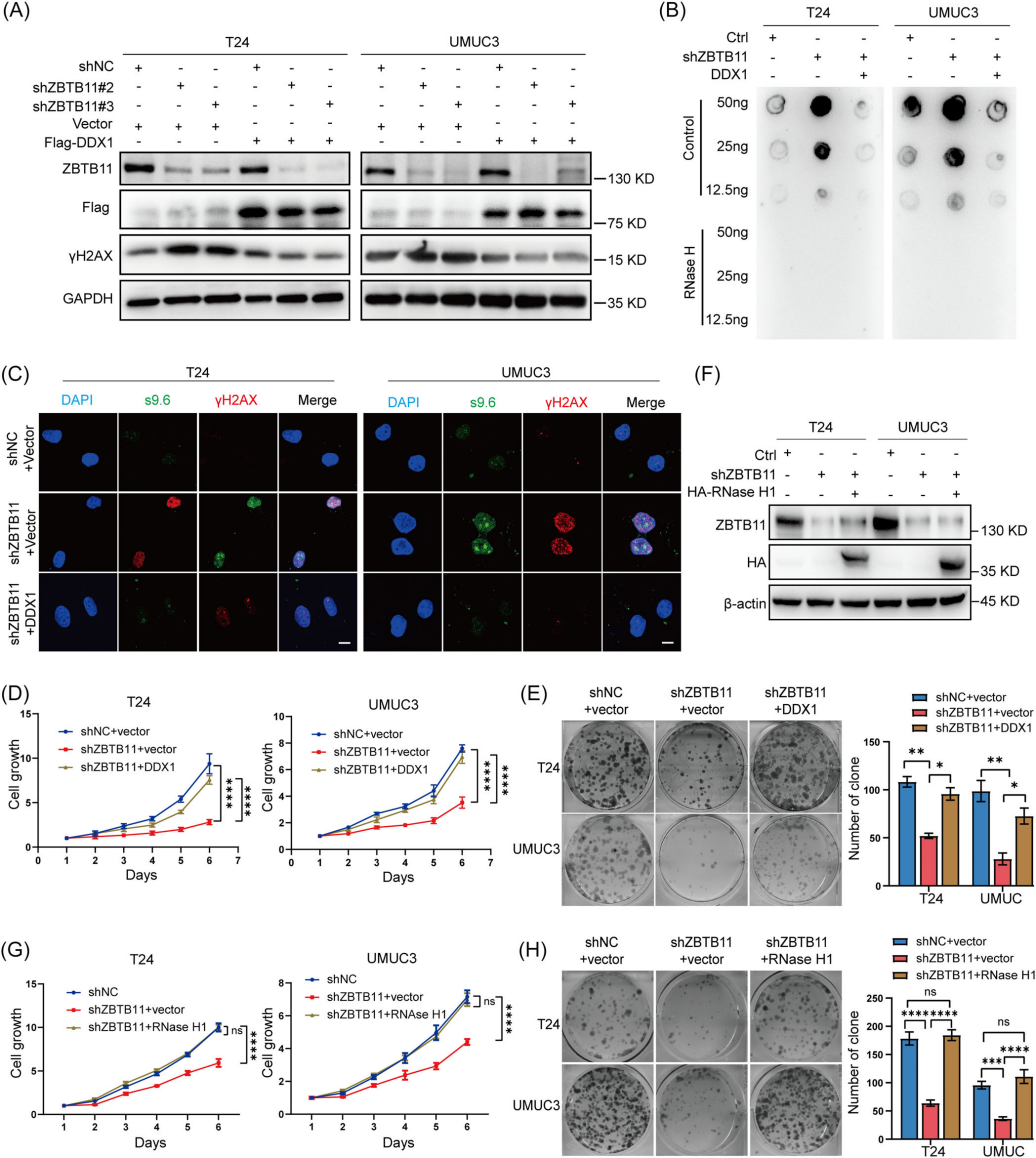
Knockdown of ZBTB11 induces R‐loop accumulation and DNA damage to suppress the growth of BC cells in a DDX1‐dependent manner. (A) Rescue of DDX1 expression in ZBTB11 knockdown BC cells was achieved by transfecting a plasmid expressing Flag‐tagged DDX1. The expressions of ZBTB11, Flag‐tagged DDX1, and γH2AX in DDX1‐rescued BC cells were detected by Western blot analysis. (B) R‐loop in ZBTB11 knockdown BC cells with DDX1 rescue was determined by Dot blot using S9.6 antibody. (C) R‐loop and DNA damage in ZBTB11 knockdown BC cells with DDX1 rescue were determined by immunofluorescence, with antibodies against S9.6 and γH2AX, respectively. Scale bar, 10 μm. (D, E) The proliferation and colony formation ability of DDX1‐rescued ZBTB11 knockdown BC cells were evaluated by MTT assay and colony formation assay, respectively. The relative cell growth curves and numbers of colonies are shown. (F) Overexpression of RNase H1 in ZBTB11 knockdown BC cells was achieved by transfecting a plasmid expressing HA‐tagged RNase H1. The expressions of ZBTB11 and HA‐tagged RNase H1 in RNase H1‐overexpressed BC cells were detected by Western blot analysis. (G, H) The proliferation and colony formation ability of RNase H1‐overexpressed ZBTB11 knockdown BC cells were evaluated by MTT assay and colony formation assay, respectively. The relative cell growth curves and numbers of colonies are shown. Statistical significance was assessed using one‐way or two‐way analysis of variance.

To determine whether the effects of ZBTB11 knockdown in BC cells were dependent on DDX1, we rescued the expression of DDX1 in ZBTB11 knockdown BC cells. Data from Western blot analysis showed that the rescue of DDX1 expression reduced γH2AX level (Figure 6A) and inhibited the R‐loop accumulation (Figure 6B,C) caused by ZBTB11 knockdown in BC cells. In addition, ZBTB11 knockdown BC cells with rescued DDX1 expression grew faster (Figure 6D,E). To test if cell apoptosis was caused by the increase in R‐loop accumulation, we overexpressed RNase H1 in ZBTB11 knockdown BC cells and found that BC cells with RNase H1 overexpression grew faster than those with ZBTB11 knockdown (Figure 6F–H). Collectively, these results demonstrate that knockdown of ZBTB11 induces R‐loop accumulation and DNA damage in a DDX1‐dependent manner to suppress the growth of BC cells.

### 3.6 The ZBTB11/DDX1 signalling axis increases sensitivity to cisplatin in BC cells

**FIGURE 7:**
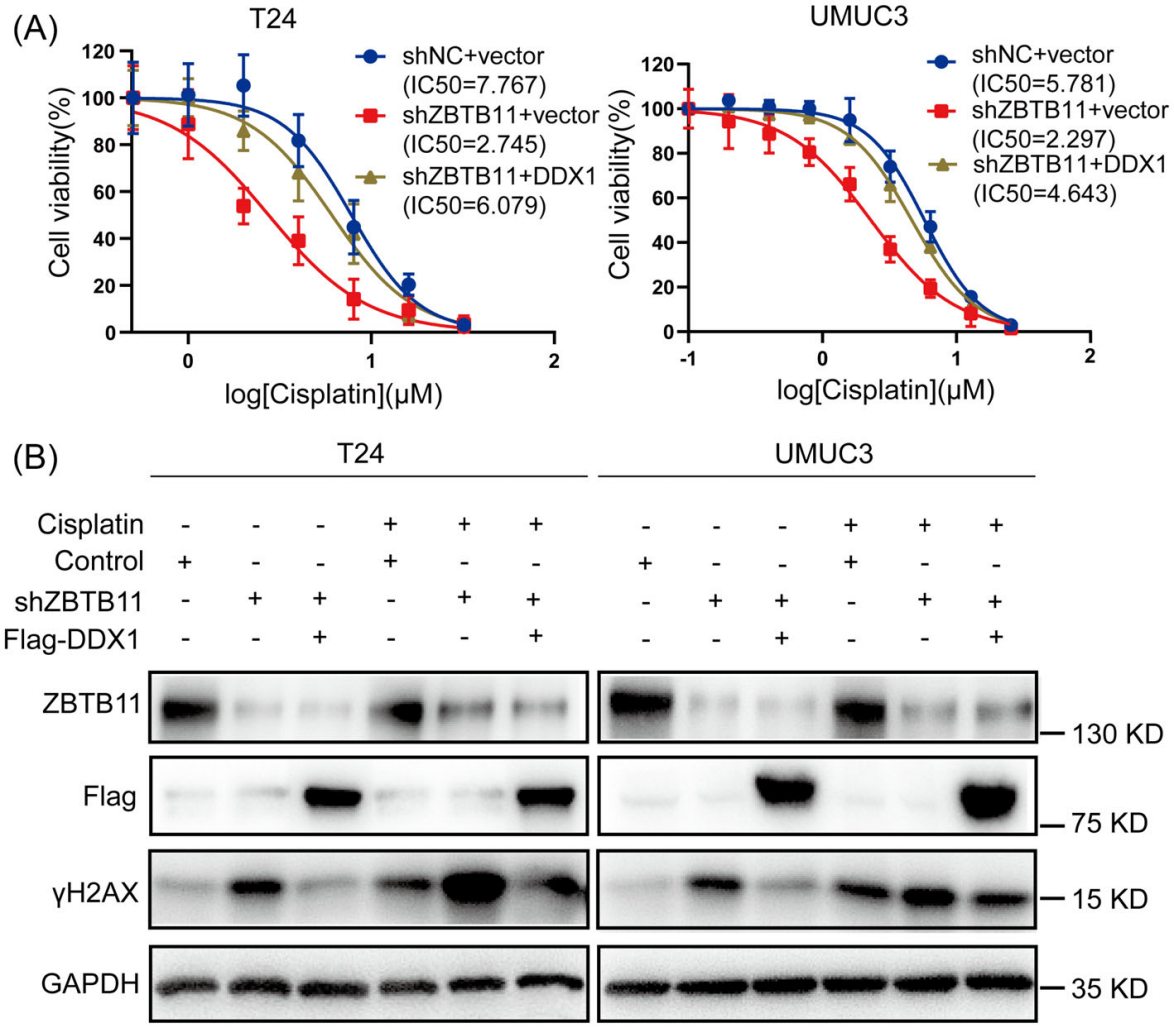
Knockdown of ZBTB11 increases sensitivity to cisplatin by downregulating DDX1 in BC cells. (A) The dose–response values of cisplatin (1–32 μM) in ZBTB11 knockdown BC cells with or without DDX1 rescue were measured using MTT assay. The IC_50_ values for cisplatin in each group were obtained from dose–response curves after fitting the data to the linear quadratic equation. (B) After the ZBTB11 knockdown BC cells with or without DDX1 rescue were treated with or without cisplatin, expressions of ZBTB11, DDX1, and γH2AX were detected by Western blot analysis.

To assess whether the ZBTB11/DDX1 signalling axis affects sensitivity to cisplatin in BC cells, we used dose–response curves to analyse the half maximal inhibitory concentration (IC_50_) values for cisplatin in ZBTB11 knockdown BC cells with or without DDX1 rescue (Figure 7A). In each group, the IC_50_ values for cisplatin showed that knockdown of ZBTB11 increased the cisplatin sensitivity of BC cells, whereas rescue of the expression of DDX1 blocked this effect (Figure 7A). Furthermore, we found that cisplatin treatment with rescued DDX1 expression reduced the increase in γH2AX levels caused by ZBTB11 knockdown in BC cells (Figure 7B). These results show that knockdown of ZBTB11 increases cisplatin sensitivity by downregulating DDX1 in BC cells

## 4 DISCUSSION

BC remains a serious health issue with high prevalence and mortality worldwide. Cisplatin is a first‐line chemotherapy for BC. However, it has poor therapeutic effect in some patients; chemotherapy resistance is a major clinical obstacle in BC treatment.^5^ This study found that overexpression of ZBTB11 is associated with poor prognosis in BC patients. We determined that ZBTB11 maintains the proliferation of BC cells by transcriptionally regulating DDX1 to accelerate R‐loop clearance; it thus inhibits DNA damage and apoptosis. The knockdown of ZBTB11 in BC cells increased their sensitivity to cisplatin by downregulating DDX1.

Most members of the ZBTB family are transcriptional suppressors. However, a minority of ZBTB proteins act as transcriptional activators, which indicates their potential for precisely regulating the expression of various genes.^34^ Several ZBTB proteins play important roles in hemopoiesis^35^ and the progression of promyelocytic leukaemia.^36^ According to data from DepMap (https://depmap.org/portal/gene/ZBTB11?tab=overview), ZBTB11 is a common essential gene and has been reported in a few articles. ZBTB11 gene inactivation leads to impaired mitochondrial respiratory function, resulting in proliferation arrest and cell death.^18^ Embryonic stem cells lacking ZBTB11 lost clonal form and exhibited reduced proliferation.^37^ All these findings support the essential role of ZBTB11 in the proliferation of normal cells. Because ZBTB11 was highly expressed in BC tissue compared with normal bladder tissue, we hypothesized that ZBTB11 may be important for BC cell proliferation and cisplatin resistance. Indeed, analysis of clinical BC samples revealed that high expression of ZBTB11 was associated with both the clinical staging and poor prognosis in BC patients, which implies that it may be a crucial transcription factor for maintaining BC tumorigenesis.

Our in vitro and in vivo investigations revealed that ZBTB11 exerts its tumorigenic function mainly through transcriptional regulation of DDX1 in BC cells. DDX1 has been widely reported to activate the transcription of oncogenes for promoting tumorigenesis in multiple tumours (e.g., testicular tumours, nephroblastoma, breast cancer, and colorectal cancer).^27^, ^28^, ^29^, ^30^ Thus, it is not surprising to find that DDX1 is necessary for BC tumorigenesis. Contrary to previous studies, we found that DDX1 could reduce DNA damage by clearing R‐loops, thereby inhibiting apoptosis of BC cells. As an RNA helicase, DDX1 can improve the efficiency of DNA repair by directly unwinding the RNA bound to DNA at double‐strand breaks.^32^, ^33^ Our findings, along with those of studies, lend plausibility to the conclusion that DDX1 silencing can promote DNA damage by increasing R‐loop accumulation.^38^, ^39^, ^40^

Because cisplatin‐based chemotherapy mainly induces double‐strand breaks and apoptosis in tumour cells, suppressing the ZBTB11/DDX1 signalling axis may increase the cisplatin sensitivity of BC cells. In fact, other inducers of R‐loop accumulation or stabilization have been used in tumour therapy (e.g., topotecan, trabectedin, and lurbinectedin).^41^, ^42^ Some studies have demonstrated that genotoxicity can enhance the chemosensitivity of tumour cells in an R‐loop‐dependent manner. For example, knockdown of THOC1 enhances sensitivity to cisplatin by increasing R‐loop formation in hepatocellular carcinoma.^25^ Moreover, the EWS‐FLI fusion protein has been shown to increase the formation of R‐loops, resulting in vulnerability to etoposide and camptothecin in Ewing's sarcoma.^43^ Based on the above evidence, inhibitors that target the ZBTB11/DDX1 signalling axis could be developed and applied to cisplatin chemotherapy in BC for improving the efficacy of chemotherapy.

In conclusion, our data revealed that ZBTB11 promotes DDX1 transcription to reduce R‐loop accumulation and DNA damage in BC cells, thus maintaining their proliferation and cisplatin resistance. We determined a novel mechanism of the tumorigenic ZBTB11/DDX1 axis in BC. We also suggested a potential target for the development of inhibitors for chemotherapy in cisplatin‐resistant patients

## AUTHOR CONTRIBUTIONS

Xianchong Zheng and Zhuowei Liu conceived the project, designed the experiments and revised the manuscript. Lei Chen, assisted by Zefu Liu, Huancheng Tang and Zhaohui Zhou performed most of the experiments, analysed the data and wrote the manuscript. Jiawei Chen, Zikun Ma, Minhua Deng, Xiangdong Li, Yuanzhong Wu, Lisi Zheng and Liwen Zhou assisted with the experiments and provided technical assistance. All authors read the final version of the manuscript and approved the submission.

## FUNDING INFORMATION

This work was supported by grants from the National Nature Science Foundation of China (NSFC) (82073103 to ZW. L. and 82103264 to XC. Z.)

## CONFLICT OF INTEREST

The authors declare no competing interests.

## Supporting information


**Supplementary Figure S1** Overexpression of ZBTB11 does not remarkably influence the growth of BC cells. (A) Overexpression of ZBTB11 in BC cells was achieved using adenovirally‐delivered Flag‐tagged ZBTB11. The expression of Flag was determined using Western blot analysis. (B) The proliferation of BC cells with ZBTB11 overexpression was evaluated by MTT assay. Relative cell growth curves are presented. Statistical significance was assessed using one‐way analysis of variance. (C) The colony formation ability of BC cells with ZBTB11 overexpression was evaluated by colony formation assay. Numbers of colonies are presented as a bar chart. Statistical significance was assessed using one‐way analysis of variance.

**Supplementary Figure S2** The expression of different genes in T24 cells analysed by RNA‐seq after ZBTB11 knockout. (A) The proliferation of T24 cells with ZBTB11 knockout was evaluated by MTT assay. Relative cell growth curves are presented. Statistical significance was assessed using one‐way analysis of variance. (B) Heat map of significantly upregulated and downregulated genes across RNA‐Seq data of ZBTB11 knockout T24 cells (*p* < 0.05; fold change > 2 or fold change < 0.5).

**Supplementary Table S1** Target sequences of shRNAs and sgRNAs
**Supplementary Table S2** Primers used in qRT‐PCR
**Supplementary Table S3** Primers used in ChIP qPCR

**Supplementary Table S4** Differential gene expression results across RNAseq data

## Data Availability

The data generated and/or analyzed during the current study are available from the corresponding author on reasonable request.
