# Supplementary figures and images for: Knockdown of ZBTB11 impedes R‐loop elimination and increases the sensitivity to cisplatin by inhibiting DDX1 transcription in bladder cancer
Source: Cell Prolif. 2022 Aug 26;55(12):e13325. doi: 10.1111/cpr.13325 (PMC9715355; doi:10.1111/cpr.13325)

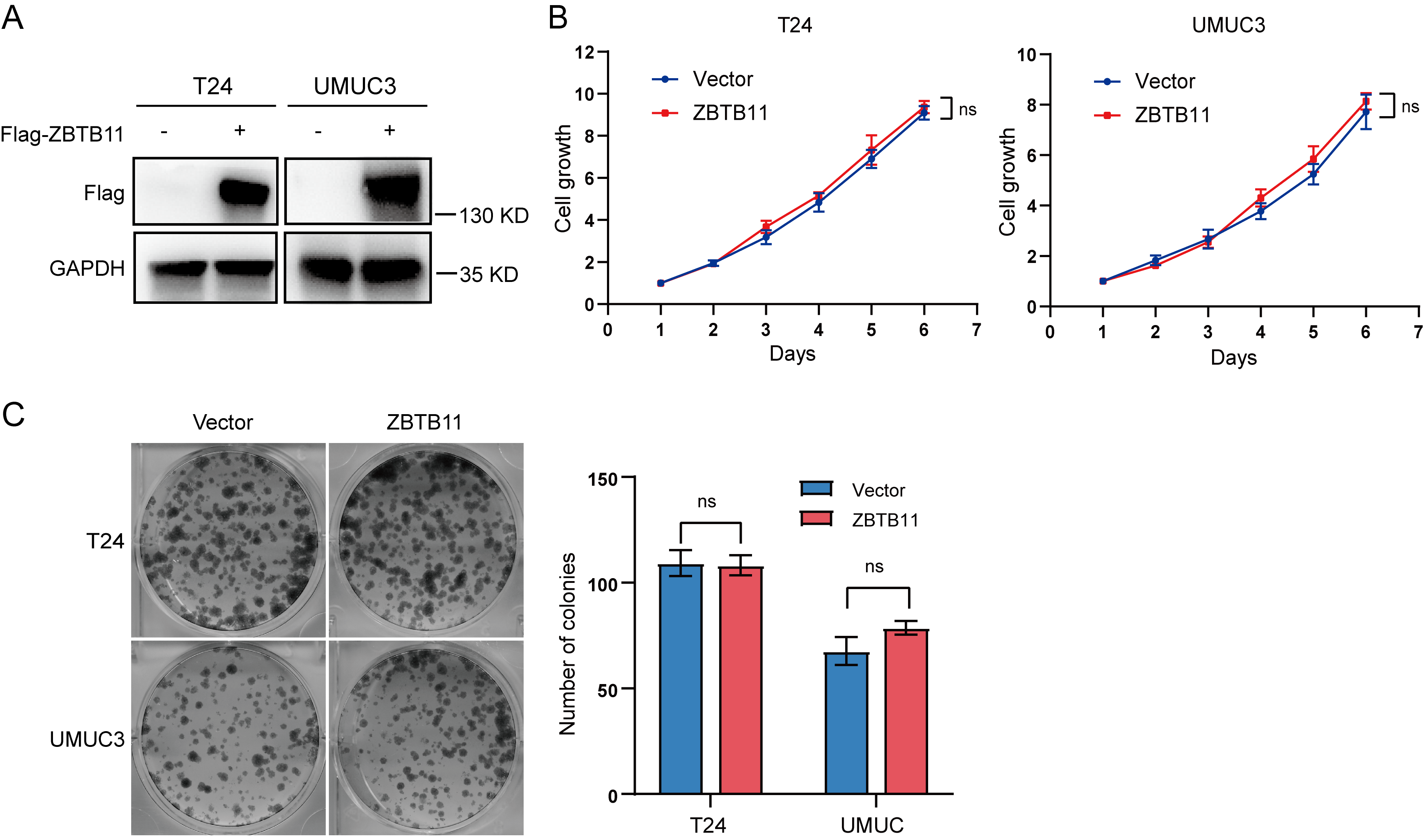

Supplement: Supplementary file 1 — Supplementary Figure S1 Overexpression of ZBTB11 does not remarkably influence the growth of BC cells. (A) Overexpression of ZBTB11 in BC cells was achieved using adenovirally‐delivered Flag‐tagged ZBTB11. The expression of Flag was determined using Western blot analysis. (B) The proliferation of BC cells with ZBTB11 overexpression was evaluated by MTT assay. Relative cell growth curves are presented. Statistical significance was assessed using one‐way analysis of variance. (C) The colony formation ability of BC cells with ZBTB11 overexpression was evaluated by colony formation assay. Numbers of colonies are presented as a bar chart. Statistical significance was assessed using one‐way analysis of variance. [file CPR-55-e13325-s004.png]

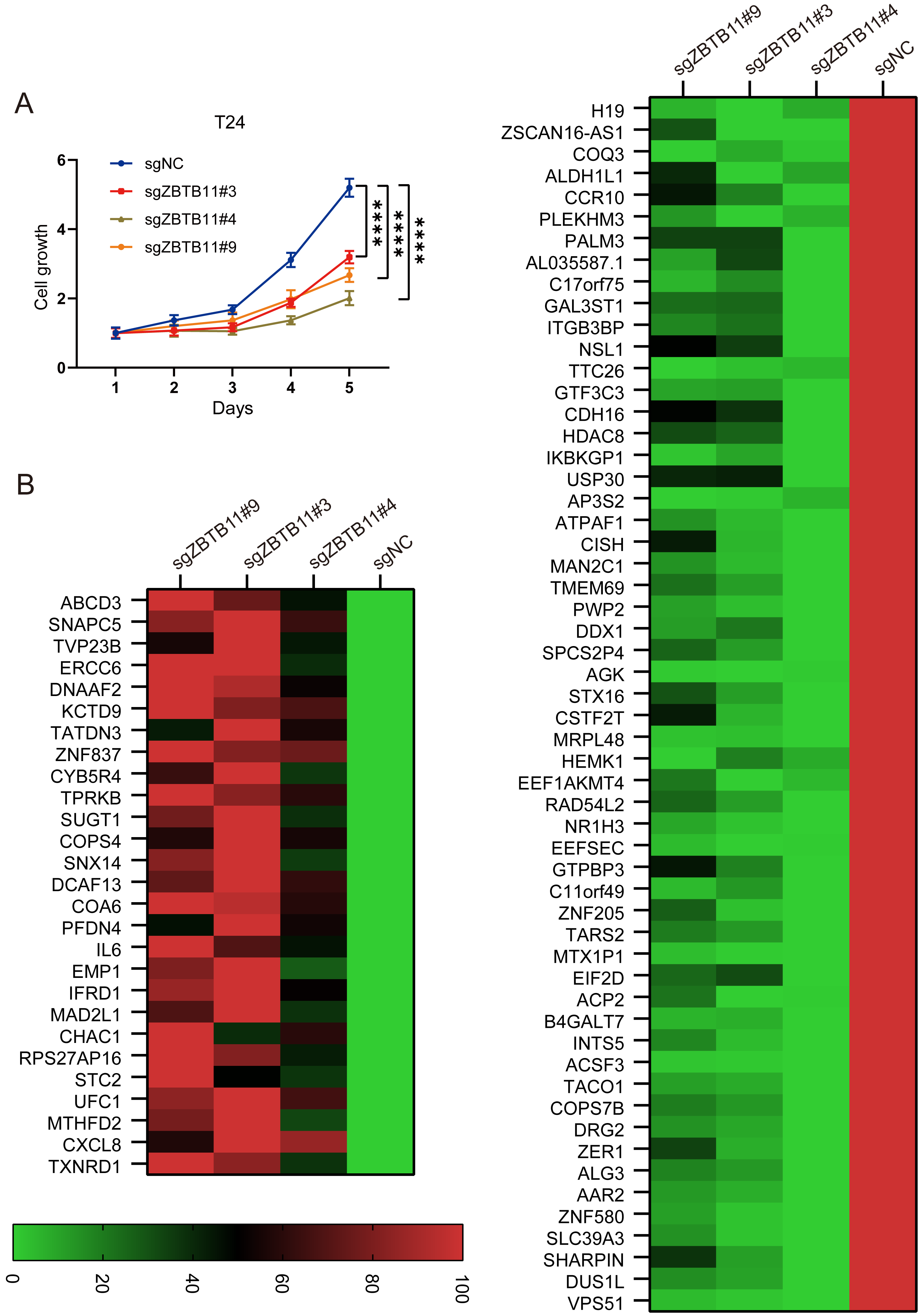

Supplement: Supplementary file 2 — Supplementary Figure S2 The expression of different genes in T24 cells analysed by RNA‐seq after ZBTB11 knockout. (A) The proliferation of T24 cells with ZBTB11 knockout was evaluated by MTT assay. Relative cell growth curves are presented. Statistical significance was assessed using one‐way analysis of variance. (B) Heat map of significantly upregulated and downregulated genes across RNA‐Seq data of ZBTB11 knockout T24 cells (p < 0.05; fold change > 2 or fold change < 0.5). [file CPR-55-e13325-s003.png]
