# Supplementary material for: Knockdown of ZBTB11 impedes R‐loop elimination and increases the sensitivity to cisplatin by inhibiting DDX1 transcription in bladder cancer
Source: Cell Prolif. 2022 Aug 26;55(12):e13325. doi: 10.1111/cpr.13325 (PMC9715355; doi:10.1111/cpr.13325)
Supplement: Supplementary file 3 — Supplementary Table S1 Target sequences of shRNAs and sgRNAs Supplementary Table S2 Primers used in qRT‐PCR Supplementary Table S3 Primers used in ChIP qPCR [file CPR-55-e13325-s001.docx]

**Supplementary Table S1** Target sequences of shRNAs and sgRNAs

| shRNA or sgRNA name | Nucleotide sequence |
| --- | --- |
| sgZBTB11#3 | GCCGCGCACCACGTAGCAGG |
| sgZBTB11#4 | GCGACGCCGGGACCTCATCG |
| sgZBTB11#9 | GACCTTCGCGGAGCTGGAGG |
| shZBTB11#2 | ATAGGATGCATAGGTATATTA |
| shZBTB11#3 | GCAGAACCAAGCGGGAATTTA |
| shDDX1#1 | GCTGTGGAAGAGATGGATTGG |
| shDDX1#3 | GGAACATAAGATGGATCAAGC |

**Supplementary Table S2** Primers used in qRT-PCR

| Primer name | Nucleotide sequence |
| --- | --- |
| ZBTB11-F | TCCTGCGTTACCTGACGAAC |
| ZBTB11-R | AGCTTTTCGGATTTTACGCTTGA |
| DDX1-F | TGGTGTCAACTGGAAAGCTG |
| DDX1-R | CAAAGTGGCAGAGCAAACAA |
| ACSF3-F | GCGCTAACGATGCCTCCTAC |
| ACSF3-R | CTGGGAGTCGCAGATGACATA |
| BCL3-F | AACCTGCCTACACCCCTATAC |
| BCL3-R | CACCACAGCAATATGGAGAGG |
| COPS7B-F | GTGTATCCCCTACTCCGTGTT |
| COPS7B-R | CATTCATGCAGGGTCTTGACA |
| DDIT3-F | GGAAACAGAGTGGTCATTCCC |
| DDIT3-R | CTGCTTGAGCCGTTCATTCTC |
| FBXO4-F | TGATCTGTGTCAGTTGGGAAGT |
| FBXO4-R | CCAAACATAGCAAATCGTGGTTC |
| GADD45A-F | GAGAGCAGAAGACCGAAAGGA |
| GADD45A-R | CACAACACCACGTTATCGGG |
| ICAM1-F | ATGCCCAGACATCTGTGTCC |
| ICAM1-R | GGGGTCTCTATGCCCAACAA |
| IL8-F | TTTTGCCAAGGAGTGCTAAAGA |
| IL8-R | AACCCTCTGCACCCAGTTTTC |
| LIG1-F | GAAGGAGGCATCCAATAGCAG |
| LIG1-R | ACTCTCGGACACCACTCCATT |
| MAD2L1-F | GTTCTTCTCATTCGGCATCAACA |
| MAD2L1-R | GAGTCCGTATTTCTGCACTCG |
| MDH1-F | GGTGCAGCCTTAGATAAATACGC |
| MDH1-R | AGTCAAGCAACTGAAGTTCTCC |
| MRPS10-F | CCAAACCTGTGGTAACAATCTCT |
| MRPS10-R | GCCTTATCGTGACCTTTCACC |
| PAX2-F | TGTCAGCAAAATCCTGGGCAG |
| PAX2-R | GTCGGGTTCTGTCGTTTGTATT |
| PMAIP1-F | ACCAAGCCGGATTTGCGATT |
| PMAIP1-R | ACTTGCACTTGTTCCTCGTGG |
| RPL26-F | GACTTCCGACCGAAGCAAGAA |
| RPL26-R | TGCACCCGTTCAATGTAGATAAC |
| SDHD-F | ATTTCTTCAGGACCGACCTATCC |
| SDHD-R | CAGCCTTGGAGCCAGAATG |
| SHARPIN-F | GGGGCGGTTAATTTGGAGTG |
| SHARPIN-R | CTCGGACTAGGACTGCCCA |
| SOX9-F | AGCGAACGCACATCAAGAC |
| SOX9-R | CTGTAGGCGATCTGTTGGGG |
| UAP1-F | AATGACCTCAAACTCACGTTGT |
| UAP1-R | GCTCTGCATAAAGTTCTACCTGT |
| GAPDH-F | TGACTTCAACAGCGACACCC |
| GAPDH-R | CTGGTGGTCCAGGGGTCTTA |
| CHIP-DDX1-F | TGGTCTTCGGCGTTCGGA |
| CHIP-DDX1-R | TCAGCGGCAGGCGCATAA |

**Supplementary Table S3** Primers used in ChIP qPCR

| Primer name | Nucleotide sequence |
| --- | --- |
| DDX1 F | TGGTCTTCGGCGTTCGGA |
| DDX1 R | TCAGCGGCAGGCGCATAA |
